# Prognostic value of the advanced lung cancer inflammation index in patients with gastric cancer after radical gastrectomy: a propensity-score matching cohort study and meta-analysis

**DOI:** 10.1186/s12885-024-12349-9

**Published:** 2024-05-13

**Authors:** Huayang Pang, Lingyan Dai, Lihui Chen, Xiufeng Chen, Zhixiong Chen, Shouru Zhang, Hao Sun

**Affiliations:** 1 Department of Gastrointestinal Cancer Center, Chongqing University Cancer Hospital, Chongqing, 400030 China; 2 Chongqing Key Laboratory of Translational Research for Cancer Metastasis and Individualized Treatment, Chongqing University Cancer Hospital, Chongqing, 400030 China; 3 School of Medicine, Chongqing University, Chongqing, 400044 China

**Keywords:** Gastric cancer, Advanced lung cancer inflammation index, Prognostic value, Propensity-score matched analysis, Meta-analysis

## Abstract

**Background:**

Insufficient evidence existed about the prognostic role of the advanced lung cancer inflammation index (ALI) for gastric cancer patients who underwent curative resection. The aim of this study was to identify the predictive ability of ALI for survival after curative gastrectomy.

**Methods:**

We retrospectively analyzed 328 gastric cancer patients who received curative gastrectomy from the database of Chongqing University Cancer Hospital, and investigated the prognostic role of the preoperative ALI compared with clinicopathological variables and other serum biomarkers, such as preoperative neutrophil-to-lymphocyte ratio (NLR), platelet to lymphocyte ratio (PLR) and Lymphocyte-monocyte ratio (LMR). To minimize intergroup differences, propensity score matching (PSM) analysis was employed. Additionally, we performed a meta-analysis of four cohort studies published up to October 2023 following the PRISMA guidelines.

**Results:**

In the overall cohort, patients in the low ALI group had a significantly worse overall survival compared to those in the high ALI group (*P* < 0.0001). Subgroup analysis identified that ALI maintained its prognostic significance across different subgroups. In addition, ROC analysis showed that ALI had a higher AUC value for 3-year overall survival compared to NLR, PLR, and LMR (0.576 vs. 0.573 vs. 0.557 vs. 0.557). Multivariate analysis indicated that ALI, other than other serum biomarkers, was an independent risk factor for decreased overall survival in GC patients following curative surgery (HR = 1.449; 95%CI: 1.028–2.045; *P* = 0.034). Consistently, PSM analysis supported all of these findings. The meta-analysis including 4 studies evaluating 2542 patients, confirmed the association between the low ALI and poor survival outcomes.

**Conclusion:**

The preoperative ALI was an independent prognostic factor for survival in gastric cancer patients who underwent curative gastrectomy.

**Supplementary Information:**

The online version contains supplementary material available at 10.1186/s12885-024-12349-9.

## Background

Gastric cancer (GC) remains the fifth most prevalent malignancy and the third leading cause of cancer-related mortality worldwide [1]. Radical gastrectomy combined with perioperative multimodal treatment represents the fundamental approach to extend long-term survival in GC patients [2]. However, despite these standardized therapies, patients’ prognosis remains unsatisfactory [3]. Therefore, it is imperative to develop treatment plans based on anticipated survival time in order to enhance curative outcomes for individuals receiving radical gastrectomy. Currently, the AJCC TNM staging system serves as the primary basis for gastric cancer management; however, relying solely on staging systems does not adequately support treatment selection and prognostic evaluation in this disease entity [4]. Consequently, there is a critical need to explore novel prognostic biomarkers that can guide therapeutic decision-making in gastric cancer.

Mounting evidence indicates that cancer-related inflammation and malnutrition prevail among the majority of patients with malignancies, exerting a pivotal influence on the prognosis of cancer patients [5, 6]. Consequently, biomarkers centered around inflammation/nutrition hold great promise as potential prognosticators for long-term oncological results. Notably, the preoperative neutrophil-to-lymphocyte ratio (NLR), obtained from two blood-based inflammatory markers, has emerged as an influential gauge linked to adverse surgical outcomes and survival rates in multiple malignancies [7]. Furthermore, reduced body mass index (BMI) and serum albumin (ALB), which mirror nutritional status, have also exhibited associations with adverse therapeutic outcomes across diverse types of cancers [8, 9].

In recent years, the advanced lung cancer inflammation index (ALI), an emerging biomarker, has emerged as a significantly more promising predictor of survival outcomes in cancers due to its incorporation of multiple nutritional and inflammatory indicators [10–12]. Specifically, the ALI is calculated as BMI * ALB/NLR, which was first established by Jafri et al. [13] in 2013 as a prognostic index for non-small cell lung cancer. Since then, the ALI has been widely applied to evaluate the prognosis of various diseases [14–16]. Our previous meta-analysis [17] revealed that a low ALI indicates poor overall survival (OS) and disease-free survival (DFS) in gastrointestinal cancer patients. However, due to limited reported studies, the prognostic role of the ALI in gastric cancer patients undergoing curative surgery has not been sufficiently investigated.

Therefore, due to insufficient evidence, we conducted a two-step study. Firstly, we carried out a retrospective cohort study to assess the predictive value of ALI for prognosis in gastric cancer patients who underwent curative gastrectomy. Secondly, we performed a meta-analysis validation using relevant studies to evaluate the prognostic role of ALI in those patients.

## Materials and methods

### Ethics statement

The present retrospective study was conducted in accordance with the principles outlined in the Declaration of Helsinki and received approval from the Research Ethics Committee of Chongqing University Cancer Hospital (ethical approval number: CZLS2023347-A). Prior to analysis, all medical records were anonymized and deidentified. Furthermore, informed consent to participate was obtained from individual patients prior to surgery in the study.

### Study population

**Fig. 1:**
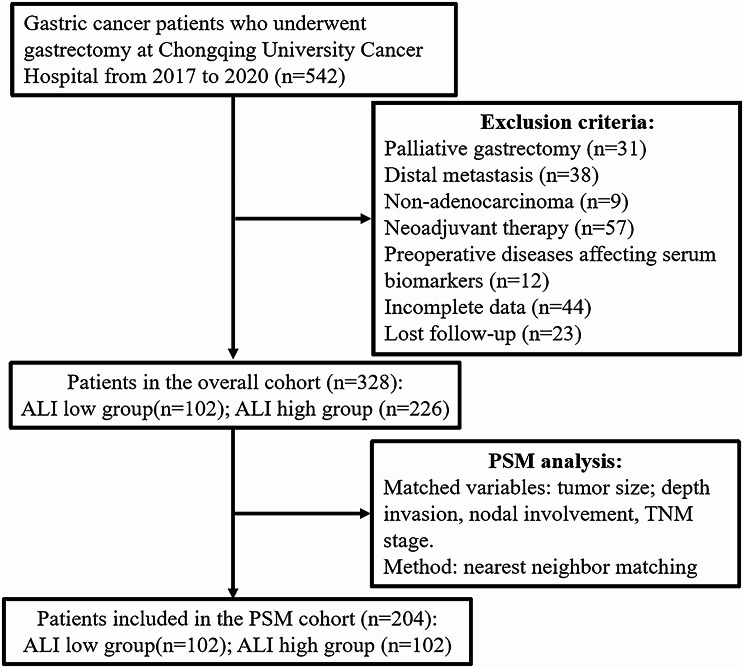
The flow chart of patients with gastric cancer enrolled in this study

A total of 542 consecutive patients with gastric cancer who underwent gastrectomy between January 2017 and June 2020 were retrieved from the database of Chongqing University Cancer Hospital for analysis in this study. Patients were included based on the following criteria: (1) histologically confirmed gastric adenocarcinoma; (2) radical resection performed; (3) absence of distant metastasis; (4) availability of complete clinical and laboratory information. Patients meeting any of the following criteria were excluded: (1) receiving neoadjuvant therapy; (2) having any inflammatory or hematological diseases affecting relevant laboratory parameters; (3) lost to follow-up. Ultimately, a total of 382 participants were included in this study (Fig. 1).

### Data collection

The following data were collected: sex, age, height, weight, comorbidities, preoperative laboratory tests (including perioperative blood cell counts and albumin levels), surgical approach, gastrectomy extent, tumor size, tumor differentiation, depth invasion, nodal metastasis, pathological stage and adjuvant chemotherapy. The perioperative venous blood was collected from all patients within one week prior to surgery for the examination of serum parameters. BMI was defined as weight (kg) divided by height-squared (m^2^). NLR was defined as absolute neutrophil count divided by absolute lymphocyte count. Platelet-lymphocyte ratio (PLR) was defined as absolute platelet count divided by absolute lymphocyte count. Lymphocyte-monocyte ratio (LMR) was defined as absolute lymphocyte count divided by absolute monocyte count. ALI was calculated as follows: ALI = BMI (kg/m^2^) × Albumin (g/dl) / NLR.

The surgery was performed in accordance with the Japanese gastric cancer treatment guidelines [18], and the tumor’s pathologic staging was conducted based on the AJCC 8th TNM system [19].

### Follow-up

The patients were regularly followed up through telephone interviews and outpatient visits. The last follow-up date in this study was June 1, 2023. It was recommended that all patients undergo follow-up every 3–6 months during the first 3 years and at least once yearly thereafter. OS time was calculated from the surgery date to the last follow-up or the date of death from any cause.

### Statistical analysis

Statistical analyses were conducted using R software (version 4.1.2; R Core Team, URL: http://www.R-project.org/) and SPSS software (IBM SPSS Statistics, Armonk, NY). Student’s t-tests were used to compare continuous variables with a normal distribution. Mann-Whitney U tests were employed for skewed continuous variables and ordinal categorical variables comparison, while chi-square tests were utilized for unordered categorical variables comparison. OS was analyzed using the Kaplan-Meier method and compared using the log-rank test. The optimal cut-off values of ALI and other serum biomarkers for OS were determined by utilizing the “surv_cutpoint” function from the “survminer” package [20]. The predictive values of these serum markers for 3-year OS were compared using the area under receiver-operating characteristic (ROC) curve (AUC). Univariate and multivariate Cox proportional hazard regression models were used to evaluate risk factors for OS, presenting results as hazard ratios (HRs) with their corresponding 95% confidence intervals (CIs). Variables with a P value < 0.10 in univariate analysis, as well as clinically significant variables, were considered as candidate variables to include in the multivariate model. We also calculated the tolerance and variance inflation factor (VIF) values to evaluate multicollinearity between these candidate variables, with tolerance < 0.1 and VIF > 10 considered indicative of multicollinearity [21]. Additionally, propensity score matching (PSM) analysis was performed to eliminate intergroup differences in baseline parameters using a 1:1 nearest matching method implemented through the “MatchIt” package [22]. All P values reported are two-tailed, and statistical significance was defined as *P* < 0.05.

### Meta-analysis

The present meta-analysis was conducted in accordance with the PRISMA guidelines. Two independent authors (P HY and D LY) comprehensively searched the online databases of PubMed, Embase, and Web of Science for potential studies from inception to October 31, 2023. The literature retrieval utilized the following combination of keywords: [“advanced lung cancer inflammatory index”] AND [“gastric cancer” OR “gastric carcinoma” OR “stomach cancer” OR “stomach tumor”]. Inclusion criteria were as follows: (1) Studies examining the association between the ALI and prognosis in GC patients who underwent primary curative resection; (2) Sufficient data provided to obtain HRs and 95% CIs for the relationship between the ALI and survival outcomes; (3) The cut-off value of the ALI was clearly reported; (4) for duplicated studies, only the study that included the most cases was included. Exclusion criteria included literature reviews, case reports, conference abstracts and letters. Data extraction was performed using a predefined form that captured study characteristics (e.g., publication year, country, sample size), clinicopathological features (e.g., age, sex, TNM stage), and survival outcomes including OS, progression-free survival (PFS), and cancer-specific survival (CSS). The risk of bias was assessed using the Newcastle Ottawa Scale [23] (NOS) for cohort studies. All pooled analyses were conducted using Review Manager Software version 5.3 (Cochrane, London UK). Heterogeneity between studies was evaluated using the I^2^ statistic; an I^2^ value greater than 50% indicated substantial heterogeneity. Pooled effects were calculated using the Mantel-Haenszel random-effects model. Statistical significance was defined as a two-tailed P-value < 0.05.

## Results

### Clinicopathological characteristics of included patients in the overall cohort

The clinicopathological features of 328 patients were summarized in Table 1 (left panel). The mean age of all patients was 58.41 ± 10.87 years old. There were 222 males (66.7%) and 106 females (32.3%). The distribution of TNM stage were as follows: 65 (19.8%) with stage I; 65 (19.8%) with stage II; 198 (60.4%) with stage III. After curative resection, adjuvant chemotherapy was administered to 56.1% of the patients. We analyzed the optimal threshold of serum biomarkers for OS using the “surv_cutpoint” function of the “survminer” package, and the optimal cut-off values of ALI, NLR, PLR and LMR were 31.90, 2.34, 164.41 and 4.55, respectively.

**Table 1:** Correlation between clinicopathological characteristics and ALI in gastric cancer patients before and after PSM analysis

Characteristics	Before PSM					After PSM			
	Total (*n* = 328)	Low ALI (*n* = 102)	High ALI (*n* = 226)	*P* value		Total (204)	Low ALI (*n* = 102)	High ALI (*n* = 102)	*P* value
**Age, years (Mean ± SD)**	58.41 ± 10.87	59.46 ± 11.08	57.93 ± 10.77	0.239		58.67 ± 11.45	59.46 ± 11.08	57.87 ± 11.80	0.482
**Age, n (%)**				0.320					0.766
≥ 65 years old	94 (28.7)	33 (32.4)	61 (27.0)			68 (33.3)	33 (32.4)	35 (34.0)	
< 65 years old	234 (71.3)	69 (67.6)	165 (73.0)			136 (66.7)	69 (67.6)	67 (68.0)	
**Sex, n (%)**				0.993					0.191
Male	222 (67.7)	69 (67.6)	153 (67.7)			129 (63.2)	69 (67.6)	60 (58.8)	
Female	106 (32.3)	33 (32.4)	73 (32.3)			75 (36.8)	33 (32.4)	42 (41.2)	
**Comorbidity, n (%)**				0.218					0.515
Yes	73 (22.3)	27 (26.5)	46 (20.4)			50 (24.5)	27 (26.5)	23 (22.5)	
No	255 (77.7)	75 (73.5)	180 (79.6)			154 (75.5)	75 (73.5)	79 (77.5)	
**Surgical approach, n (%)**				0.917					0.622
Open	33 (10.1)	10 (9.8)	23 (10.2)			18 (8.8)	10 (9.8)	8 (7.8)	
Laparoscopic	295 (89.9)	92 (90.2)	203 (89.8)			186 (91.2)	92 (90.2)	94 (92.2)	
**Gastrectomy extent, n (%)**				0.276					0.058
Proximal	49 (14.9)	20 (19.6)	29 (12.8)			29 (14.2)	20 (19.6)	9 (8.8)	
Distal	186 (56.7)	54 (52.9)	132 (58.4)			109 (53.4)	54 (52.9)	55 (53.9)	
Total	93 (28.4)	28 (27.5)	65 (28.8)			66 (32.4)	28 (27.5)	38 (37.3)	
**Tumor size, cm (Mean ± SD)**	5.57 ± 3.15	6.05 ± 2.84	5.36 ± 3.26	0.003		6.16 ± 3.06	6.05 ± 2.84	6.27 ± 3.28	0.624
**Tumor size, n (%)**									0.877
≥ 5 cm	189 (57.6)	73 (71.6)	116 (51.3)	0.001		145 (71.1)	73 (71.6)	72 (70.6)	
< 5 cm	139 (42.4)	29 (28.4)	110 (48.7)			59 (28.9)	29 (28.4)	30 (29.5)	
**Tumor differentiation, n (%)**				0.404					0.086
Differentiated	99 (30.2)	34 (33.3)	65 (28.8)			57 (27.9)	34 (33.3)	23 (22.5)	
Undifferentiated	229 (69.8)	68 (66.7)	161 (71.2)			147 (72.1)	68 (66.7)	79 (73.5)	
**Depth invasion, n (%)**				0.019					0.245
T1	54 (16.5)	8 (7.8)	46 (20.4)			20 (9.8)	8 (7.8)	12 (11.8)	
T2	43 (13.1)	14 (13.7)	29 (12.8)			31 (15.2)	14 (13.7)	17 (16.7)	
T3	62 (18.9)	20 (19.6)	42 (18.6)			40 (19.6)	20 (19.6)	20 (19.6)	
T4	169 (51.5)	60 (58.8)	109 (48.2)			113 (55.4)	60 (58.8)	53 (52.0)	
**Nodal metastasis, n (%)**				0.186					0.126
N0	88 (26.8)	19 (18.6)	69 (30.5)			35 (17.2)	19 (18.6)	16 (15.7)	
N1	48 (14.6)	17 (16.7)	31 (13.7)			36 (17.6)	17 (16.7)	19 (18.6)	
N2	63 (19.3)	25 (24.5)	38 (16.8)			35 (17.2)	25 (24.5)	10 (9.8)	
N3	129 (39.3)	41 (40.2)	88 (38.8)			98 (48.0)	41 (40.2)	57 (55.9)	
**TNM stage, n (%)**				0.038					0.870
I	65 (19.8)	11 (10.8)	54 (23.9)			24 (11.8)	11 (10.8)	13 (12.7)	
II	65 (19.8)	23 (22.5)	42 (18.6)			42 (20.6)	23 (22.5)	19 (18.6)	
III	198 (60.4)	68 (66.7)	130 (57.5)			138 (67.6)	68 (66.7)	70 (68.7)	
**Adjuvant chemotherapy, n (%)**				0.594					0.480
Yes	184 (56.1)	55 (53.9)	129 (57.1)			115 (56.4)	55 (53.9)	60 (58.8)	
No	144 (43.9)	47 (46.1)	97 (42.9)			89 (43.6)	47 (46.1)	42 (41.2)	
**BMI, Kg/m** ^**2**^ **(Mean ± SD)**	22.1 ± 3.19	20.61 ± 2.82	22.77 ± 3.13	< 0.001		21.73 ± 3.26	20.61 ± 2.82	22.85 ± 3.31	< 0.001
**Albumin, g/L (Mean ± SD)**	40.18 ± 4.51	38.38 ± 5.09	40.99 ± 3.98	< 0.001		39.46 ± 4.60	38.38 ± 5.09	40.54 ± 3.79	< 0.001
**Neutrophil count, 10** ^**9**^ **/L (Mean ± SD)**	3.45 ± 1.30	4.29 ± 1.59	3.06 ± 0.93	< 0.001		3.60 ± 1.45	4.29 ± 1.59	2.91 ± 0.85	< 0.001
**Lymphocyte count, 10** ^**9**^ **/L (Mean ± SD)**	1.55 ± 0.54	1.20 ± 0.40	1.70 ± 0.53	< 0.001		1.41 ± 0.49	1.20 ± 0.40	1.62 ± 0.47	< 0.001
**Monocyte count, 10** ^**9**^ **/L (Mean ± SD)**	0.32 ± 0.13	0.34 ± 0.17	0.31 ± 0.11	0.067		0.32 ± 0.14	0.34 ± 0.17	0.30 ± 0.10	0.163
**Platelet count, 10** ^**9**^ **/L (Mean ± SD)**	194.21 ± 73.66	207.81 ± 86.97	188.07 ± 66.09	< 0.001		197.16 ± 76.42	207.81 ± 86.97	186.50 ± 62.80	0.222
**NLR (Mean ± SD)**	2.47 ± 1.35	3.80 ± 1.64	1.88 ± 0.54	< 0.001		2.83 ± 1.55	3.80 ± 1.64	1.87 ± 0.54	< 0.001
**ALI (Mean ± SD)**	44.51 ± 22.75	23.06 ± 6.39	54.20 ± 20.76	< 0.001		38.40 ± 21.82	23.06 ± 6.39	53.75 ± 20.98	< 0.001
**PLR (Mean ± SD)**	137.38 ± 69.02	185.19 ± 90.11	115.80 ± 42.13	< 0.001		152.53 ± 77.17	185.19 ± 90.11	119.87 ± 41.19	< 0.001
**LMR (Mean ± SD)**	5.41 ± 2.45	4.12 ± 2.06	5.99 ± 2.39	< 0.001		4.92 ± 2.05	4.12 ± 2.06	5.73 ± 1.71	< 0.001

PSM: propensity-score matched analysis; SD: standard difference; BMI: body mass index; ALI: advanced lung cancer inflammation index; NLR: neutrophil to lymphocyte ratio; PLR: platelet to lymphocyte ratio; LMR: lymphocyte to monocyte ratio

Overall, a total of 102 patients were identified as having a low ALI (< 31.90), and 226 patients were identified as having a high ALI (≥ 31.90). We observed that a low ALI was significantly associated with larger tumor size, more advanced tumor depth invasion, and higher TNM stage. Additionally, we found that individuals with a low ALI exhibited lower BMI, albumin levels, lymphocyte count, LMR level but higher neutrophil count, monocyte count, platelet count, NLR level and PLR level.

### Survival analyses according to the ALI in the overall cohort

**Fig. 2:**
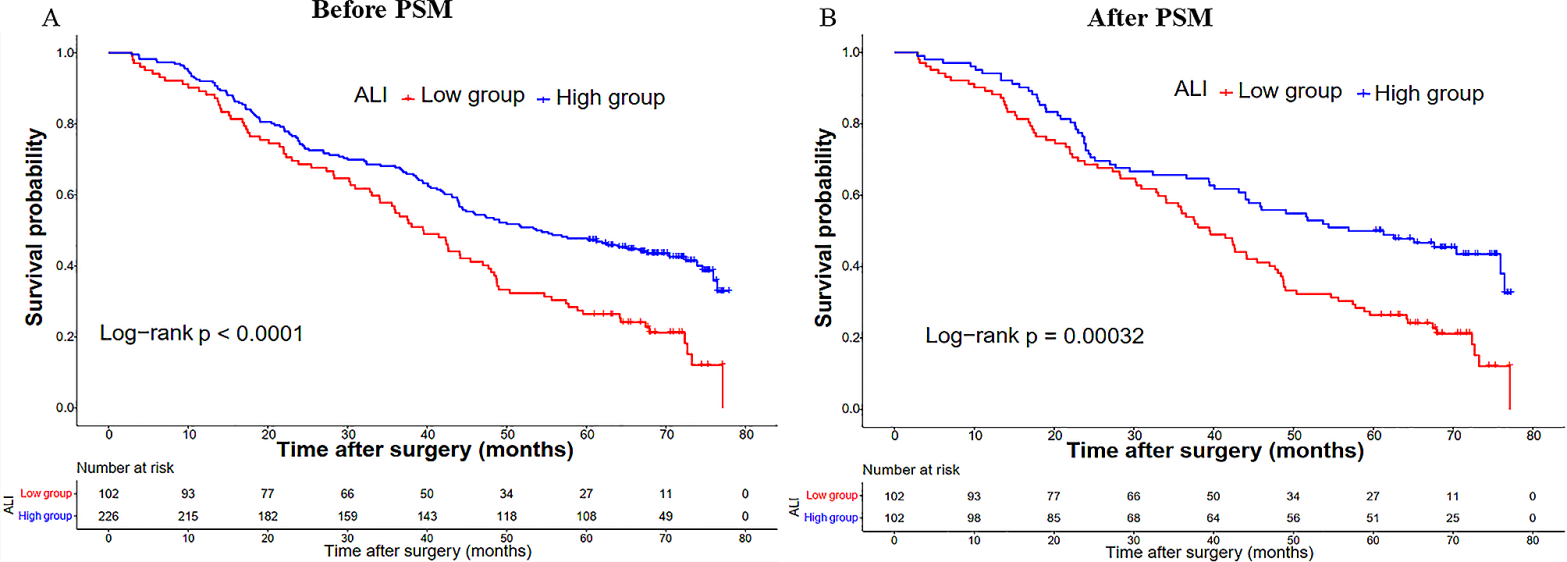
Kapan-Meier curves of ALI in the overall (**A**) and PSM (**B**) cohorts

**Fig. 3:**
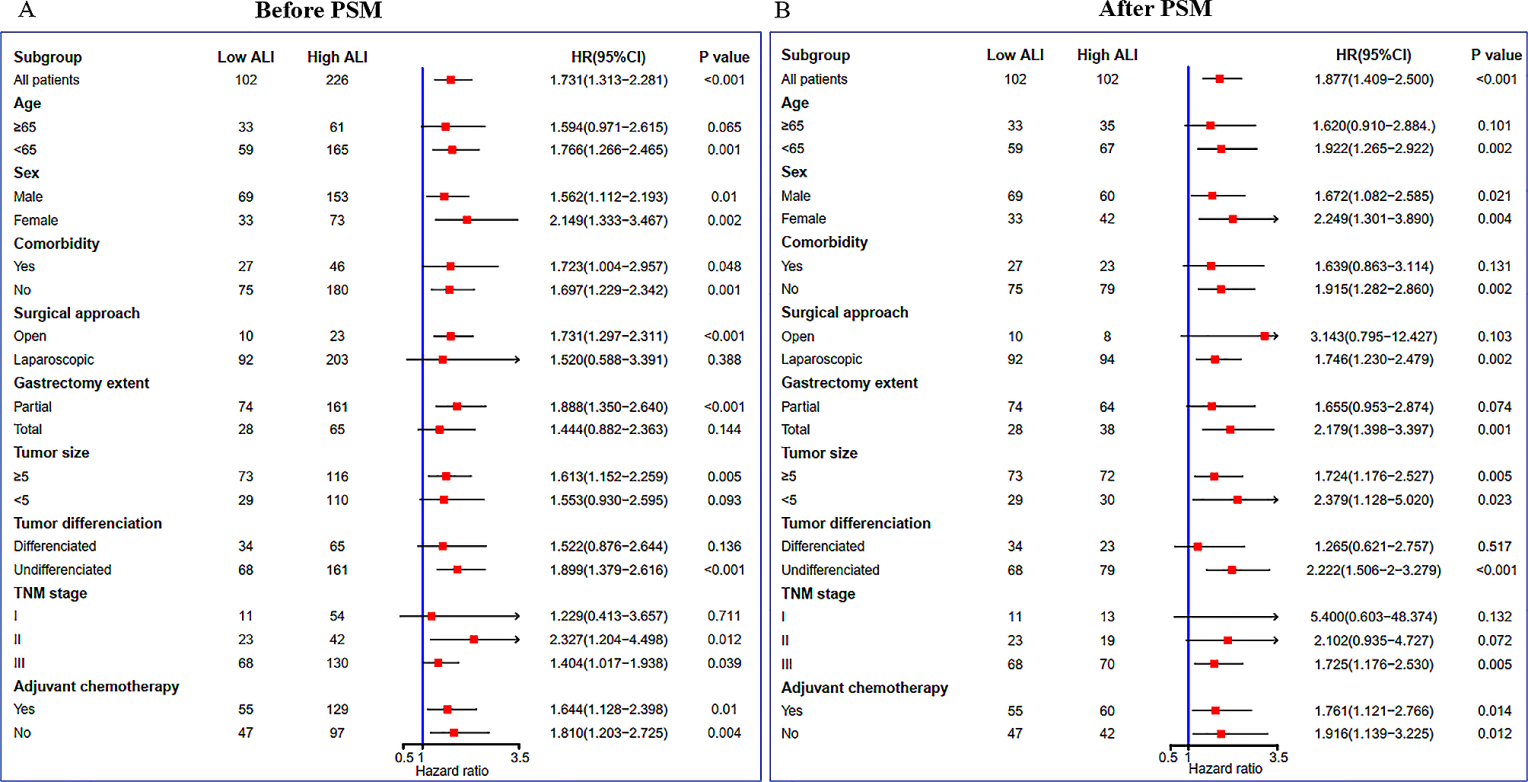
Subgroup analyses of ALI for overall survival in the overall (**A**) and PSM (**B**) cohorts

In the overall cohort, the Kaplan-Meier survival curve demonstrated a significantly worse OS in patients belonging to the low ALI group compared to those in the high ALI group (3-year OS rate: 54.9% vs. 67.7%; *P* < 0.0001, Fig. 2A). Furthermore, subgroup analyses based on various clinicopathologic features were conducted to investigate the prognostic value of ALI in different types of gastric cancer patients. As shown in Fig. 3A, our findings identified that ALI maintained its prognostic significance across different subgroups.

**Fig. 4:**
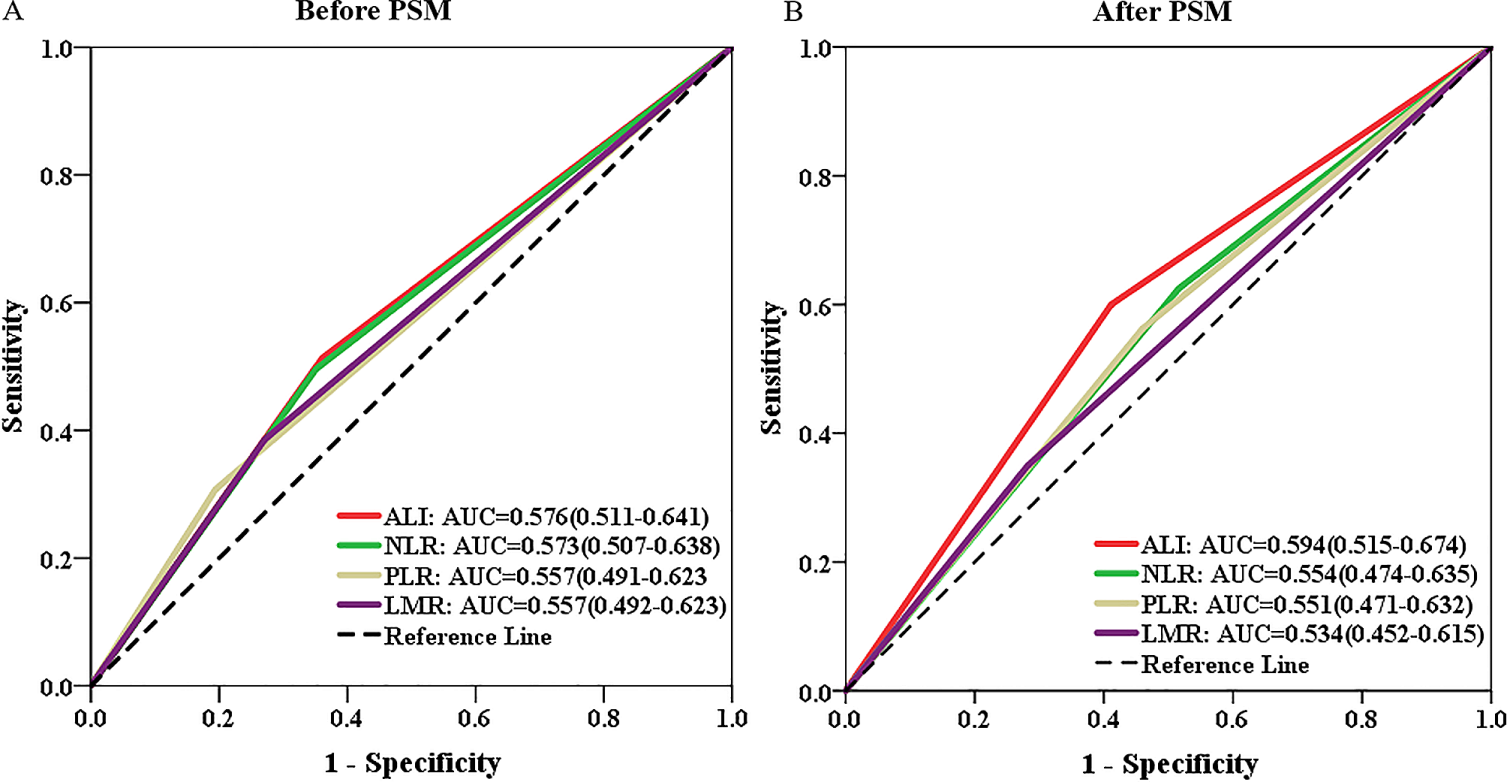
Predictive abilities of ALI and other hematological indices for 3-year overall survival examined using t-ROC curves in the overall (**A**) and PSM (**B**) cohorts

In order to compare the prognostic predictability of ALI with other hematological biomarkers, we generated ROC curves and calculated their corresponding AUCs. In the overall cohort, the AUC for 3-year OS was higher for ALI compared to NLR, PLR, and LMR (0.576 vs. 0.573 vs. 0.557 vs. 0.557) (Fig. 4A).

In the overall cohort, the univariate analysis revealed significant associations of ALI, preoperative comorbidity, gastrectomy extent, tumor differentiation, TNM stage, LMR and PLR with overall survival (all P values < 0.05). In the multivariate analysis, candidate covariates were identified as variables with a significance level of *P* < 0.1 and adjuvant chemotherapy, which were further confirmed to have no collinearity among independent variables (all candidate variables had tolerance values > 0.1 and VIF values < 10, as shown in Table S1). We identified that the low ALI independently posed a risk factor for decreased OS (HR = 1.449; 95%CI: 1.028–2.045; *P* = 0.034; Table 2).

**Table 2:** Univariable and multivariable analysis for predictors of overall survival in the overall cohort

	Univariate analysis			Multivariate analysis	
	HR (95% CI)	*P* value		HR (95% CI)	*P* value
Age, years					
≥ 65 vs. <65	1.070 (0.754–1.517)	0.424			
**Sex**					
Male vs. Female	1.056 (0.751–1.485)	0.827			
**Comorbidity**					
Yes vs. No	1.641 (1.137–2.367)	0.011		1.300 (0.944–1.791)	0.108
**Surgical approach**					
Laparoscopic vs. Open	0.533 (0.278–1.023)	0.132			
**Gastrectomy extent**					
Total vs. Partial	1.499 (1.065–2.111)	0.001		1.286(0.954–1.735)	0.099
**Tumor size, cm**					
≥ 5 vs. <5	1.760 (1.185–2.614)	< 0.001		1.091 (0.790–1.504)	0.600
**Tumor differentiation**					
Undifferentiated vs. Differentiated	1.438 (0.977–2.118)	0.001		1.342 (0.960–1.875)	0.085
**TNM stage**					
II vs. I	1.730 (1.130–2.646)	0.010		**2.079 (1.410–3.067)**	**< 0.001**
III vs. I	5.376 (2.353–12.195)	< 0.001		**3.846 (1.626–5.882)**	**< 0.001**
**Adjuvant chemotherapy**					
Yes vs. No	0.827 (0.592–1.153)	0.329		**0.731 (0.552–0.969)**	**0.030**
**ALI**					
Low vs. High	1.844 (1.314–2.586)	< 0.001		**1.449 (1.028–2.045)**	**0.034**
**LMR**					
Low vs. High	1.648 (1.181–2.299)	0.009		1.161 (0.852–1.585)	0.343
**PLR**					
Low vs. High	0.742 (0.525–1.048)	0.003		1.070 (0.749–1.526)	0.711

HR: hazard ratio; CI: confidence interval; ALI: advanced lung cancer inflammation index; PLR: platelet to lymphocyte ratio; LMR: lymphocyte to monocyte ratio

### Sensitivity analysis according to PSM analysis

To mitigate baseline bias, a 1:1 PSM analysis was conducted, resulting in 102 patients in each group. Following the matching process, there were no statistically significant differences observed in the clinicopathological parameters between the two groups (Table 1, right panel). Following PSM, patients in the low ALI group continued to exhibit inferior OS (3-year OS rate: 54.9% vs. 64.7%; *P* = 0.00032, Fig. 2B). As depicted in Fig. 3B, our findings consistently demonstrated that ALI maintained its prognostic value across different subgroups after PSM. Similarly, in the PSM cohort, the ALI demonstrated superior prognostic prediction efficacy compared to NLR, PLR, and LMR (0.594 vs. 0.554 vs. 0.551 vs. 0.534, Fig. 4B).

### Meta-analysis validation

The literature search identified a total of 304 studies. After screening titles, abstracts, and full-texts, four retrospective single-center studies [24–27] were ultimately included (Figure S1). As depicted in Tables 3 and 4, a total of 2542 patients with gastric cancer who underwent primary curative gastrectomy were encompassed in these studies. These investigations were conducted by researchers from China and Japan and published between 2020 and 2023, with sample sizes ranging from 358 to 949. Among the included studies, the cut-off values of ALI ranged from 24.81 to 40.50. All of the incorporated studies reported OS, two studies reported PFS, and one study reported CSS. Furthermore, all the studies underwent assessment using the NOS checklist and achieved an NOS score of seven stars, indicating their satisfactory quality (Table 3 and Table S2). However, due to only four included studies being available for analysis, publication bias assessment was not performed in this meta-analysis.

**Table 3:** Basic information of included studies

Reference	Country	Study design	Study interval	Sample size, *n*	Age, years	Sex	TNM stage	Exclusion of disease affecting biomarker examination	Time of biomarker detection	NOS score
Chen,2023	China	R; S	2008–2020	949	60(20–97)	615/334	I-III	Yes	2 weeks before surgery	7
He,2022	China	R; S	2009–2014	358	61(56–67)	284/74	I-II	Yes	1 week before surgery	7
Yin,2020	Japan	R; S	1992–2011	620	NR	424/196	I-IV	Yes	2 weeks before surgery	7
Zhang,2022	China	R; S	2010–2017	615	NR	469/146	I-III	Yes	NA	7

R: retrospective; S: single center; NA: not available; NOS: Newcastle Ottawa Scale

**Table 4:** Survival information of included studies

Reference	cut-off method	cut-off value	Sample size, *n*		Follow-up time, months	Survival analysis	Multivariate analysis	OS: HR (95% CI)	CSS; PFS: HR (95% CI)
			Low ALI	High ALI					
Chen,2023	X-tile	24.81	156	793	35	OS; CSS	Yes	1.55(1.11–2.16)	1.46(1.01–2.10)
He,2022	ROC	40.50	116	242	101 (range, 2–166)	OS	Yes	1.338(0.735–2.436)	NA
Yin,2020	ROC	30	171	449	52.8 ± 39.9	OS; PFS	Yes	1.59(1.15–2.19)	1.26(0.51–3.11)
Zhang,2022	ROC	39.77	253	362	NA	OS; PFS	Yes	1.34(1.02–1.73)	1.36(1.04–1.79)

ALI: advanced lung cancer inflammation index; OS: overall survival; PFS: progression free survival; CSS: cancer specific survival; HR: hazard ratio; CI: confidence interval; NA: not available

**Fig. 5:**
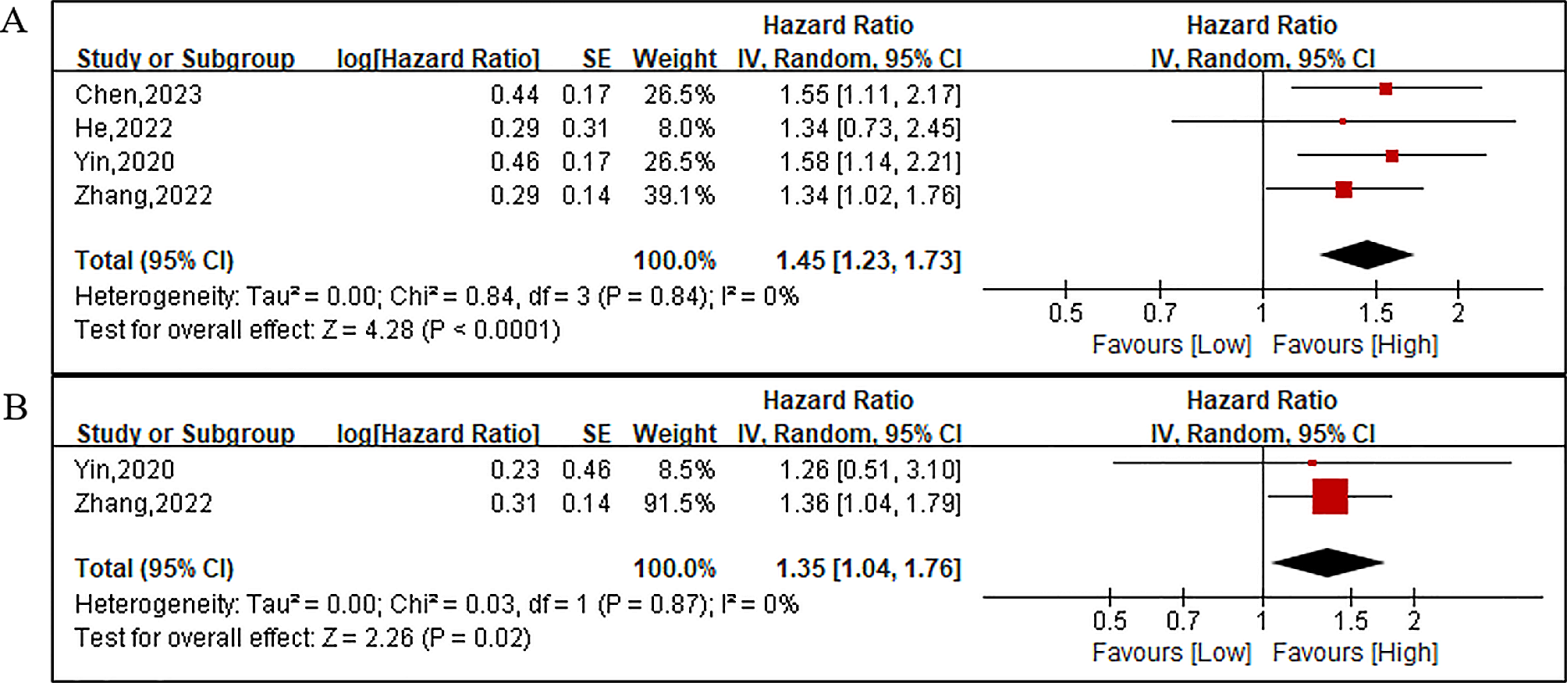
Forest plots of overall survival (**A**) and progression-free survival (**B**) between the low ALI and high ALI groups

The analysis included four studies with a total of 2542 patients (696 in the low ALI group and 1846 in the high ALI group) reporting on OS. The pooled HR was 1.45 (95%CI: 1.23–1.73; *P* < 0.0001; I^2^ = 0%; Fig. 5A), indicating that a low ALI independently increased the risk of deteriorated OS. Two studies involving 1235 patients (424 in the low ALI group and 811 in the high group) reported on PFS. The combined results revealed that patients with a low ALI had significantly worse PFS compared to those with a high ALI (HR = 1.35; 95%CI:1.04–1.76; *P* = 0.02; I^2^ = 0%; Fig. 5B). Additionally, one study investigated the association of ALI with CSS, demonstrating that ALI could serve as a valuable serum biomarker for predicting CSS in gastric cancer patients undergoing primary curative surgery (HR = 1.46; 95%CI:1.01–2.10; *P* = 0.043).

## Discussion

As a novel and simple serum biomarker, the ALI has been widely investigated to be a potential prognostic biomarker in multiple malignancies except for lung cancer [28], such as colorectal cancer [14], endometrial cancer [29] and neuroblastoma [30]. Nevertheless, few studies [24–27] are available on the prognostic value of ALI in gastric cancer. Hence, in the present study, we collected and analyzed the data for GC patients at our center and conducted a meta-analysis to draw a more definitive conclusion.

We retrospectively analyzed 328 GC patients after curative resection and examined the prognostic factors potentially affecting OS, such as ALI, LMR, PLR and various clinicopathological characteristics. Our study showed that the ALI, but not other inflammatory biomarkers, such as LMR and PLR, independently predicted OS in both overall and PSM cohorts. In addition, subgroup analyses supported the prognostic predictability of ALI in different types of GC patients. Furthermore, ROC analyses revealed that ALI had a higher AUC value to predict 3-year OS rate compared to other biomarkers like NLR, PLR and LMR before and after PSM analysis. Overall, the findings of our cohort study supported the prognostic superiority of ALI in GC patients who received curative resection.

To further explore the clinical utility of ALI in GC curative surgery, we conducted a comprehensive literature search followed by meta-analysis validation. To our knowledge, this is the first meta-analysis to explore the prognostic value of ALI in GC patients who underwent curative gastrectomy. Consistent with our cohort study, the pooled analysis revealed that the preoperative ALI was an independent prognostic factor for OS in in those patients, with a low heterogeneity. In addition, our meta-analysis also found that the low preoperative ALI was an independent risk factor for poor PFS and CSS. Therefore, ALI may serve as a valuable tool for predicting long-term survival outcomes in this part of GC patients.

Dysregulation of systemic inflammation and malnutrition of the host has been identified as being involved in the tumor progression [4]. Simultaneously, a lower ALI may result from lower BMI, ALB, lymphocytes, and higher neutrophils. Although the detailed mechanisms of the ALI’s prognostic value in cancers are unclear, they can be explained as follows: Firstly, the baseline BMI and ALB, which serve as objective and universally recognized indicators of patients’ nutritional status, have consistently been reported to be associated with clinical outcomes of cancer patients [31, 32]. Kim et al. [33] found that a low preoperative BMI had a detrimental impact on DFS and OS in patients with stage I/II gastric cancer, and was associated with a higher incidence of major complications in patients with stage III/IV gastric cancer. A study by Ouyang et al. [34] also demonstrated that a low preoperative ALB was related to decreased OS in GC patients who underwent surgical treatment. In addition, a low serum albumin has been confirmed to prompt the production of pro-inflammatory cytokines and reduce cell-mediated immunity [35]. Second, as a well-established inflammation-related biomarker, the NLR has been extensively validated to predict long-term oncological outcomes in various malignancies [7]. A recent umbrella review of systematic reviews and meta-analyses has demonstrated a positive association between higher NLR and an increased HR for survival outcomes in cancer patients [36]. The underlying mechanism lies in the ability of neutrophils to create a conducive microenvironment for tumor cell proliferation, as well as their role in promoting tumor cell progression and invasion [37]. Conversely, a decrease in lymphocyte counts hampers the immune response against cancer cells [38]. The presence of lymphopenia has been shown to be correlated with an unfavorable prognosis in individuals diagnosed with cancer [39]. Consequently, the ALI, in conjunction with these factors, serves as a valuable comprehensive indicator of nutritional and inflammatory status, facilitating a deeper comprehension of patients’ functional state and offering predictive insights into survival outcomes for individuals with gastric cancer.

The present study has several limitations that should be acknowledged. Firstly, our dataset was collected retrospectively, with a limited sample size. Additionally, the studies included in the meta-analysis were also designed to be retrospective nature. Consequently, there is a potential risk of selection bias that cannot be ignored, necessitating further investigation through prospective studies. Secondly, the participants of both our study and those included in the meta-analysis were solely from China and Japan; therefore, the absence of studies from other regions may restrict the generalizability of our findings. Lastly, it is worth noting that there was variation in the cut-off value for ALI across different cohorts (ranging from 24.81 to 40.50), which could potentially impact its validity and applicability in clinical practice.

## Conclusions

The findings of the present study demonstrate that preoperative ALI, an easily measurable biomarker associated with host inflammation and nutrition, holds significant predictive value for survival outcomes in gastric cancer patients who underwent curative gastrectomy. However, further validation of ALI’s utility in this population necessitates high-quality prospective studies with large sample sizes.

### Electronic supplementary material

Below is the link to the electronic supplementary material.

Supplementary Material 1

## Data Availability

The datasets used and/or analyzed during the current study are available from the corresponding author on reasonable request.

## Abbreviations

GC: Gastric cancer
NLR: Neutrophil-to-lymphocyte ratio
BMI: Body mass index
ALB: Albumin
ALI: Advanced lung cancer inflammation index
PLR: Platelet-lymphocyte ratio
LMR: Lymphocyte-monocyte ratio
OS: Overall survival
DFS: Disease-free survival
PFS: Progression-free survival
CSS: Cancer-specific survival
ROC: Receiver-operating characteristic
HR: Hazard ratio
CI: Confidence interval
PSM: Propensity score matching
NOS: Newcastle Ottawa Scale
