# Supplementary material for: Prognostic value of the advanced lung cancer inflammation index in patients with gastric cancer after radical gastrectomy: a propensity-score matching cohort study and meta-analysis
Source: BMC Cancer. 2024 May 13;24:583. doi: 10.1186/s12885-024-12349-9 (PMC11089784; doi:10.1186/s12885-024-12349-9)
Supplement: Supplementary file 1 — Supplementary Material 1 [file 12885_2024_12349_MOESM1_ESM.docx]

**Figure S1. The PRISMA flow chart of study selection.**


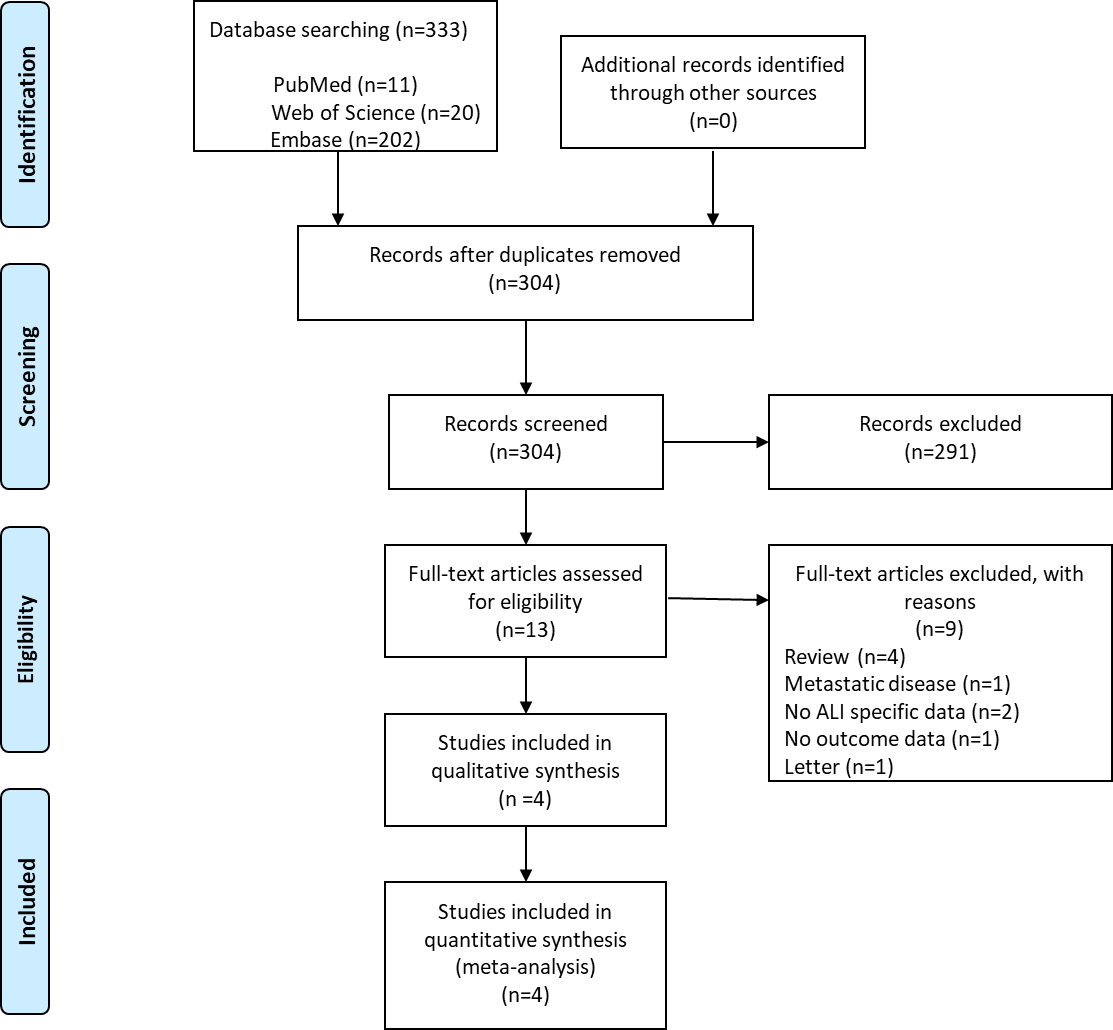


**Table S1.** **The tolerance and VIF for the overall cohort, ALI is regarded as the dependent variable, and the remaining influencing factors are independent variables.**

| **Variables** | **tolerance** | **VIF** |
| --- | --- | --- |
| Comorbidity | 0.957 | 1.045 |
| Gastrectomy extent | 0.851 | 1.175 |
| Tumor size | 0.691 | 1.447 |
| Tumor differentiation | 0.901 | 1.110 |
| TNM stage | 0.669 | 1.496 |
| Adjuvant chemotherapy | 0.944 | 1.059 |
| LMR | 0.757 | 1.321 |
| PLR | 0.819 | 1.222 |

ALI: advanced lung cancer inflammation index; PLR: platelet to lymphocyte ratio; LMR: lymphocyte to monocyte ratio; VIF: variance inflation factor.

**Table S2. Literature quality assessment using Newcastle Ottawa Scale.**

| **Cohort study** | **Representatives of the exposed cohort** | **Selection of the non-exposed cohort** | **Ascertainment of exposure** | **Was outcome of interest present at start of study** | **Comparability of cohorts on the basis of the design or analysis** | **Assessment of outcome** | **Was follow-up long enough for outcomes to occur** | **Adequate follow up** | **Total** |
| --- | --- | --- | --- | --- | --- | --- | --- | --- | --- |
| Chen,2023 | 1 | 1 | 1 | 0 | 0 | 1 | 1 | 1 | 7 |
| He,2022 | 1 | 1 | 1 | 0 | 0 | 1 | 1 | 1 | 7 |
| Yin,2020 | 1 | 1 | 1 | 0 | 0 | 1 | 1 | 1 | 7 |
| Zhang,2022 | 1 | 1 | 1 | 0 | 0 | 1 | 1 | 1 | 7 |
